# Personalized medicine in colorectal cancer diagnosis and treatment: a systematic review of health economic evaluations

**DOI:** 10.1186/s12962-018-0085-z

**Published:** 2018-01-22

**Authors:** Annamaria Guglielmo, Nicoletta Staropoli, Monica Giancotti, Marianna Mauro

**Affiliations:** Department of Clinical and Experimental Medicine, “Magna Græcia” University, Viale Europa 88100, Catanzaro, Italy

**Keywords:** Personalized medicine, Economic evaluation, Cost-effectiveness analysis, Colorectal cancer, CRC

## Abstract

**Background:**

Due to its epidemiological relevance, several studies have been performed to assess the cost-effectiveness of diagnostic tests and treatments in colorectal cancer (CRC) patients.

**Objective:**

We reviewed economic evaluations on diagnosis of inherited CRC-syndromes and genetic tests for the detection of mutations associated with response to therapeutics.

**Methods:**

A systematic literature review was performed by searching the main literature databases for relevant papers on the field, published in the last 5 years.

**Results:**

20 studies were included in the final analysis: 14 investigating the cost-effectiveness of hereditary-CRC screening; 5 evaluating the cost-effectiveness of KRAS mutation assessment before treatment; and 1 study analysing the cost-effectiveness of genetic tests for early-stage CRC patients prognosis. Overall, we found that: (a) screening strategies among CRC patients were more effective than no screening; (b) all the evaluated interventions were cost-saving for certain willingness-to-pay (WTP) threshold; and (c) all new CRC patients diagnosed at age 70 or below should be screened. Regarding patients treatment, we found that KRAS testing is economically sustainable only if anticipated in patients with non-metastatic CRC (mCRC), while becoming unsustainable, due to an incremental cost-effectiveness ratio (ICER) beyond the levels of WTP-threshold, in all others evaluated scenarios.

**Conclusions:**

The poor evidence in the field, combined to the number of assumptions done to perform the models, lead us to a high level of uncertainty on the cost-effectiveness of genetic evaluations in CRC, suggesting that major research is required in order to assess the best combination among detection tests, type of genetic test screening and targeted-therapy.

**Electronic supplementary material:**

The online version of this article (10.1186/s12962-018-0085-z) contains supplementary material, which is available to authorized users.

## Background

Personalized medicine (PM) tailors medical treatment to a patient’s personal history, genomic profile and/or specific biomarkers, with one of the most important approaches relies on scientifically developed correlations between responses to medications and specific genetic variants. PM has the potential to better respond to the increasing burden of chronic disease and the complexity of co-morbidities in term of sustainability of healthcare systems [1]. Indeed, the increasing weight of chronic diseases, such as cancer, on the demand for healthcare services and on the infrastructures required to support them, represents one of the major global health problems of the twenty-first century in term of sustainability of the whole system [2]. On these bases, the development of chronic disease management system based on PM approach should be prioritized globally, especially because it may offer, at the same time, new challenges for financial sustainability and new opportunities for industry and national economies [1, 3, 4]. Several studies demonstrated that a PM approach offers different potential benefits such as reduction of adverse drug reactions (ADRs), improvements of patients’ treatment adherence and better clinical and economic outcomes [1, 5, 6]. Furthermore, additional economic benefits would be gained by limiting the prescription and reimbursement of drugs only to patients who are most likely to respond to treatment.

Due to its epidemiological importance, several studies have been performed in the field of economic evaluations in PM for the diagnosis and treatment of patients with colorectal cancer (CRC), the third most common cancer worldwide. Of note, approximately 25% of newly diagnosed patients experience a metastatic disease ab initio and almost 50% of all CRC patients will develop metastases over time, contributing to a high mortality rate. Hence, CRC imposes a substantial healthcare cost burden on individual patients and society. CRC is a multifactorial disease, with inheritance component accounting for approximately 6% of all patients. Lynch syndrome (LS) is the most common cause of hereditary CRC. Early detection of LS, provides an opportunity for a preventive cancer approach. Additionally, genetic mutations make some tumors less responsive to specific treatments. In this scenario, the stratification of patients into genetic subgroups for targeted therapies represented an efficacious strategy in improving treatments’ clinical effects. Thus, summarizing, in the view of personalized medicine, molecular characterization in CRC hit the natural history of this disease at different time point: (i) may help in the identification of predisposing conditions; (ii) in advanced CRC permitted the transition from conventional cytotoxic drugs to molecular biomarkers-driven decision for the selection of most suitable biologic agents, with improvement in survival endpoints and safety; (iii) may improve the identification of specific prognostic subgroups.

Beyond the clinical benefits, they significantly influenced the economic impact of the treatment due to the increasing use of target therapy [5]. Thus, starting from their clinical efficacy, an assessment of economic value should consider their combined impact.

Economic evaluations are widely used in healthcare system, especially for the reimbursement of pharmaceuticals in those sectors where the continuous rising of costs undermines the sustainability of the whole system [7–9]. The main aim of evaluations of a genetic testing and/or its associated therapy is to compare differences in costs to differences in health effects between alternative therapies providing support to the decision process. Indeed, these kind of evaluations suggest whether both the information generated from the diagnostic test and the expected outcomes from the targeted therapy justify their costs [10]. In this scenario, cost-effectiveness analyses (CEAs) of pharmacogenomics profiling appeared of utmost importance.

This work aims to review the economic evidence supporting diagnosis of inherited syndromes associated with CRC and evaluation of genetic tests for the detection of specific mutations associated with response to therapeutics in metastatic CRC (mCRC). Our review considers adherence to the best practice modelling guidelines as well as the assumptions made in CRC models relating to specific aspects of the disease. Our analysis also provide a summary of the findings of the previous economic evaluations in the investigated field, that health providers, policy and decision makers should take into account for a better organization of National Health Systems (NHSs).

## Materials and methods

### Eligibility criteria

For inclusion in our review, studies had to meet all the following criteria:

1. *Subjects* healthy individuals, CRC patients undergoing evaluation for LS disease, CRC patients with genetic mutation, CRC patients undergoing treatment for metastatic disease;
2. *Intervention* any genetic test used for LS diagnosis or for predicting treatment response to anti-EGFR monoclonal antibodies in CRC;
3. *Study type* decision-analytic models, economic evaluations, including methods, input data and results, model or trial-based, CEA, cost-utility-analysis (CUA) or cost–benefit-analysis (CBA).

Specifically, for Lynch syndrome, we included any intervention (including combinations) related to strategies to identify LS in the population, strategies to manage LS in the population and strategies to manage patients in whom LS is identified. For mCRC, we extended our research to all economic evaluations on any genetic test for the detection of specific mutations associated with response to therapeutics in mCRC.

### Search strategy

A systematic literature review of PubMed, Web of Science and the Cochrane Library was conducted in order to identify all economic evaluation studies published in the period 2011–2016 on genetic mutation tests for CRC; furthermore, a backward citation chasing on included studies has been performed to eventually include additional research paper. We chose a 5-years horizon to be relevant to current practice, taking into account that (1) KRAS testing has been approved and included in American and European guideline between 2009 and 2010; (2) the FDA approved the TheraScreen kit in 2012; (3) the first NGS (which has revolutionized the speed and throughput of cataloguing such cancer-related genomic alterations) based studies of CRC genomes has been performed between 2011 and 2012. The search strategy combined terms related to economic evaluation, colorectal cancer and neoplasms. We included other terms such as cost analysis, cost–benefit, economics and genetic therapy. Search filters were used to limit the searches to economic studies related to humans as appropriate. Systematic reviews, if identified, were excluded although their bibliographies were searched for potentially includable studies. No restrictions were initially placed on the language of the articles; however, any studies not reported in the English language were excluded from the review during screening. Data were reported according to the PRISMA statement [11].

The electronic search strategy for Pubmed is included in Additional file 1.

### Study selection

After identification of publications by electronic databases, duplicated records were removed.

Papers were first screened on title and abstract and were excluded when one or more of the eligibility criteria were not met. Two reviewers, independently, screened for relevant search results. Full-text inclusion assessment, data extraction and quality assessment were conducted by one reviewer and checked by a second. Disagreement between the reviewers were resolved by discussion. After the reviewers had completed the screening process, the bibliographies of included papers were scrutinised for further potentially includable studies. All studies meeting inclusion criteria were reviewed and information on design and model characteristics, incremental cost-effectiveness ratios (ICERs), and conclusions were then extracted and summarized. We also evaluated the adherence of models to the health economic modelling report guidelines: the Consolidated Health Economic Evaluation Reporting Standards (CHEERS) [12].

### Data extraction

Findings from the included studies were extracted by one reviewer (AG) into pre-developed evidence tables, in order to extract the main characteristics of the selected health economic evaluations (e.g. type of economic evaluation, model type, time horizon, cycle length, target population, comparators, outcome measure, perspective of analysis, time horizon, type of sensitivity analysis). To facilitate comparisons all values expressed in different currencies were converted to Euro values in price year 2016 via purchasing power parities (PPPs), as proposed by Welte et al. [13] and Drummond et al. [14]. PPP values were retrieved from the OECD [15] (see Additional file 2).

## Results

**Fig. 1:**
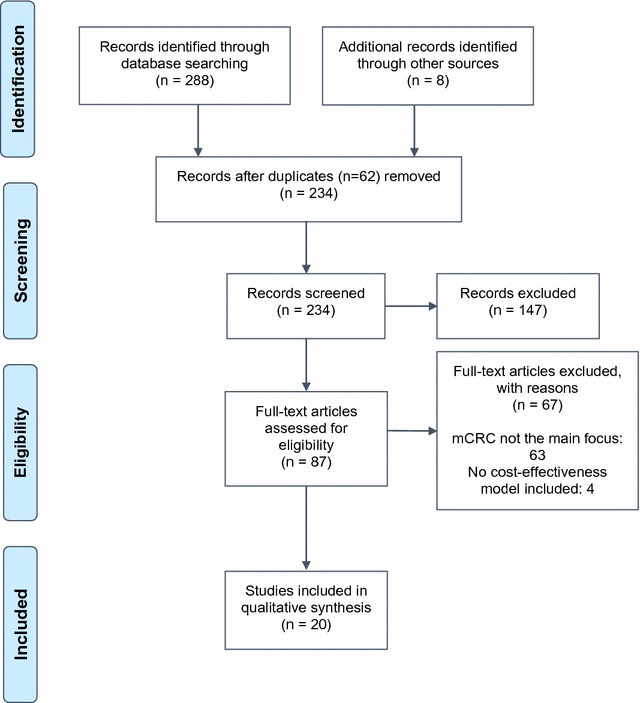
Preferred reporting items for systematic reviews and meta-analyses flow diagram [11]; mCRC: metastatic Colorectal Cancer

The search strategy identified 296 publications, with 62 duplicated being removed. Among records screened, 147 were excluded after title and abstract screening because not matching inclusion criteria. Of the remaining 87, after full text review, only 20 have been identified that have met the inclusion criteria and were included in the systematic review (Fig. 1) [16].

All studies vary in terms of perspectives, population analysed, country settings and assumptions. Table 1 outlines some of the general study characteristics. A detailed analysis is provided in Additional file 3.

**Table 1:** Main characteristics of the included studies

Study	Country	Population	Type of test
Alberts et al. [36]	USA	CRC patients with stage II, T3, MMR-P	Oncotype DX (12-gene assay)
Barone et al. [34]	IT	High-risk non-mCRC patients	KRAS mutation testing
Barzi et al. [17]	USA	Healthy individuals	IHC, Amsterdam criteria, MMR, PREMM, germline, BRAF, MSI, RBG
Behl et al. [33]	USA	Patients with mCRC	KRAS and BRAF mutation testing
Blank et al. [31]	CH	Patients with mCRC who are chemorefractory	KRAS and BRAF mutation testing
Dinh et al. [27]	USA	Population aged 20 years or over	Genetic sequencing and rearrangement; single-site testing; IHC; MSI
Gallego et al. [18]	USA	CRC patients with genetic mutations	NGS; IHC; BRAF V600E
Gausachs et al. [19]	NR (*Probably ES*)	CRC patients with genetic mutations	BRAF V600E, MLH1 promoter hypermethylation
Gould-Suarez et al. [20]	USA	CRC patients	RBG, BRAF targeted mutation analysis, IHC; MSI, MMR gene sequencing
Gudgeon et al. [21]	USA	CRC patients	IHC staining for the 4 MMR proteins, MSI, BRAF mutation, MMR gene sequencing/rearrangement analyses (Seq-Rearr), and methylation of the MLH1 promoter. (tumor testing and genetic testing)
Ladabaum et al. [28]	USA	CRC patients and their relatives	Amsterdam, IHC, BRAF, MSI, MMR, PREMM, RBG
Leenen et al. [22]	DE	CRC patients	MSI, IHC, MMR gene sequencing, RBG
Severin et al. [23]	DE	CRC patients and their relatives	IHC, MSI, BRAF V600 mutation, genetic sequencing, MMR, Amsterdam criteria, RBG
Sie et al. [24]	DE	CRC patients and their relatives	IHC, MSI, MLH1, germline
Snowsill et al. [26]	UK, Wal	CRC patients aged under 50 years and their FDRs	MSI, IHC, BRAF V600E, Amsterdam criteria
Snowsill et al. [25]	UK	CRC patients aged under 50 years	IHC, MSI, BRAF
Vijayaraghavan et al. [32]	USA, DE	Patients with mCRC in 2nd-line treatment	KRAS mutation testing
Wang et al. [29]	USA	CRC patients (at a mean age of 48 years in the base case) and their relatives (at a mean age of 25 years)	IHC, BRAF, MSI, RBG, Amsterdam criteria, MMRpredict, MMRpro, PREMM, germline
Wang et al. [30]	SG	21-year-old FDRs of mutation-confirmed LS cases	NR
Westwood et al. [35]	UK, Wal	Adult mCRC whose metastases are confined to liver and are unresectable	KRAS mutation testing

*USA* United States, *IT* Italy, *CH* Switzerland, *ES* Spain, *DE* Germany, *UK* United Kingdom, *Wal* Wales, *SG* Singapore, *CRC* Colorectal cancer, *mCRC* metastatic Colorectal cancer, *MMR-P* mismatch-repair-proficient, *LS* Lynch syndrome, *FDRs* first-degree relatives, *NR* not reported, *NGS* next-generation-sequencing, *RBG* Revised Bethesda Guidelines

Studies were divided into three main categories: Hereditary (14 studies)—KRAS (5 studies)—prognosis (1 study).

### Hereditary

All the 14 studies included in this category were further grouped as follows:

- Those that looked at the short-term cost-effectiveness of identifying LS (diagnosis). Specifically, this subgroup includes 10 studies and represents the largest subgroup of all selected studies [17–26]. Among these studies:
- 5 focused on strategies to identify LS in CRC patients regardless of age [18–21, 23];
- 4 focused on strategies to identify LS in CRC patients with pre-specified age cut-offs [22, 24–27];
- 1 focused on healthy individuals (in order to prevent CRC) [17];
- Those that examined the long-term impact on cost-effectiveness of both the strategies to identify and manage LS (diagnosis and management). This subgroup includes 4 studies [27–30].

Table 2 presents a summary of the main outcome measures of the included studies.

**Table 2:** Outcomes measures

Study	Health outcomes	Measurement of effectiveness	WTP^a^
Alberts et al. [36]	QALY	Cost saving	47,438 €/QALY
Barone et al. [34]	QALY	ICER	60,000 €/QALY
Barzi et al. [17]	LYG	ICER	47,438 €/LYG
Behl et al. [33]	LYG	ICER	98,876 €/QALY
Blank et al. [31]	QALY	ICER	47,438–98,876 €/QALY (USA); 23,342–35,014 €/QALY (UK)
Dinh et al. [27]	QALY/LYG	ICER	47,438 €/QALY
Gallego et al. [18]	QALY	ICER	98,876 €/QALY
Gausachs et al. [19]	Incremental increase in N. of cases detected	ICER	NR
Gould-Suarez et al. [20]	Incremental increase in N. of cases detected	ICER	NR
Gudgeon et al. [21]	Incremental increase in N. of cases detected	ICER	NR
Ladabaum et al. [28]	LYG	ICER	47,438 €/LYG
Leenen et al. [22]	LYG	ICER	40,000 €/LYG
Severin et al. [23]	LYG	ICER	50,000 €/LYG
Sie et al. [24]	LYG	ICER	80,000 €/LYG
Snowsill et al. [27]	QALY	ICER	23,342 €/QALY
Snowsill et al. [25]	QALY	ICER	NR
Vijayaraghavan et al. [32]	LYG	ICER	NR
Wang et al. [29]	QALY	ICER	NR
Wang et al. [30]	LYG	ICER	NR
Westwood et al. [35]	QALY	ICER	19,841 €/QALY

*QALY* quality-adjusted life year, *WTP* willingness-to-pay, *ICER* incremental cost-effectiveness ratio, *LYG* life years gained, *USA* United States, *UK* United Kingdom, *NR* not reported

^a^Currencies transformed into 2016 Euro values via purchasing power parities (PPPs)

#### LS diagnosis

- *LS diagnosis: strategies to identify LS in CRC patients* These studies investigated whether genetic screening strategies among CRC patients may be a cost-effective approach for the diagnosis of LS. Gudgeon et al. demonstrated that routine LS screening program with immunohistochemistry (IHC) followed by BRAF represents the most cost-effective option [21]. These results were also confirmed by others [21, 27–29]. Gould-Suarez et al. found that parallel testing strategies, applying IHC and MSI simultaneously as initial screening, have similar cost-effectiveness compared to sequential testing that starts with IHC and favorable cost-effectiveness compared to sequential testing that starts with MSI [20]. Finally, with a considerable drop in cost, universal germline testing may become the most cost-effective strategy for the diagnosis of LS in the CRC population. Severin et al. reported that all screening strategies reduce cancer incidence and death and yield more life-years than no-screening strategy [23]. However, LS screening provides clinical benefit but at a high cost. The most cost-effective strategy involves family-history (FH) assessment with the Revised-Bethesda-Guidelines (RBG), followed by IHC testing, BRAF testing and genetic sequencing [17, 23]. The results by Gallego et al. confirm that sequencing LS genes alone by next generation sequencing (NGS), at its current price, is not cost-effective as compared with current standard (IHC followed by MLH1, BRAF), but if others genes associated with high-penetrance CRCP syndromes were added to the panel, the intervention became cost-effective [18]. Indeed, the use of extended NGS panels, being able to detect multiple syndromes, as first-line test, can translate into better health outcomes than alternatives and likely provide acceptable value to the healthcare system. Gausachs et al. found that somatic hypermethylation of MLH1 is an accurate and cost-effective pre-screening method in patients affected by CRC with mismatch repair (MMR) deficiency and positive FH, for the selection of patients that are candidates for MLH1 germline analysis when LS is suspected and MLH1 protein expression is absent [19]. In general, most of the studies found that screening strategies among CRC patients are more effective for the diagnosis of LS than no screening strategies [18–21]. However, among these studies, there were mixed results for which screening strategy were most cost-effective, depending on model assumptions and costs included. Furthermore, it should be noted that several results depends on ICERs based on cost-per-case detected (not on LYG or QALYs of patients), which could not be compared to a measure of cost-effectiveness [19–21]. Among this subgroup, only one study showed the cost-effectiveness of screening strategies based on FH compared with no testing and with other screening strategies [23]. On these bases, results do not indicate which individual test is the most cost-effective [25].
- *LS diagnosis: strategies to identify LS in CRC patients with pre*-*specified age cut*-*offs* A further point investigated by several authors was the economic evaluation of routine LS screening among CRC patients in specific age ranges. In line with previous studies [24, 25, 28], Leenen et al. showed that routine screening for LS in CRC patients ≤ 70 years by analysis of MSI, IHC, and MLH1 hypermethylation was cost-effective [22]. Additionally, its work suggested that age-targeted LS screening might be much easier and more cost-effective to implement in clinical practice than clinical criteria based on FH. Moreover, LS screening without any age cut-off could further increase benefit for LS carrier. However, it is unclear whether the benefit of universal LS screening will come at acceptable costs. In this regard, Barzi et al. assessed the no cost-effectiveness of universal tumor testing for LS [17], proving that the combination with predictive models was cost-effective, but only in the case of available FH, according with other studies [23]. As previously reported, Sie et al. showed that testing for LS all CRC patients ≤ 70 years was more cost-effective than using an age limit of 50 years, including family cascade screening [22, 24–28]. In 2014, Snowsill et al. investigated cost-effectiveness of routine LS screening among newly diagnosed patients ≤ 50 years, assessing that all strategies compared with no testing have an ICER ≤ 11,671 €/QALY [25]. The most cost-effective strategy was based on MSI test followed by BRAF testing and genetic testing, with an ICER of 6408 €/QALY while the maximum health benefit would be obtained using universal germline testing. When the age limit was progressively raised from 50 to 70 years, the ICERs increased but remained below willingness-to-pay (WTP) (except for universal germline testing ≤ 70 years). Results suggest that reflex testing for LS in newly diagnosed CRC patients aged ≤ 50 years is cost-effective. Such testing may also be cost-effective in newly diagnosed CRC patients aged ≤ 60 or ≤ 70 years [22, 24, 28]. On these bases, Snowsill et al. in 2015 developed a model to estimate the cost-utility of strategies to identify LS in early-onset CRC (≤ 50 years) (probands) and their relatives, who would be offered predictive genetic testing if a LS mutation was found in a proband [28]. Results showed that at a WTP threshold of 23,342 €/QALY, MSI followed by BRAF followed by diagnostic genetic testing resulted in the greatest health benefit. All strategies have been considered cost-effective vs no testing [22, 24, 25, 28]. In conclusion, all studies agree that testing for LS in all new CRC patients diagnosed at age 70 or below, is more effective than current practice using an age limit of 50 years and that diagnostic mutation tests would not be cost-effective versus strategies with preliminary test.
- *Healthy individuals* (*in order to prevent CRC*) Barzi et al. investigated cost-effectiveness between proband [28] vs general population [27] screening, suggesting how a combination strategy using IHC and prediction models in probands was more cost-effective, but only in the case of available FH, according to other results [17, 23]. Furthermore, clinical criteria and general population screening strategies for LS did not emerge as cost-effective approaches. These results were not consistent with those by Dihn et al., where screening individuals with a predicted-risk of carrying LS of 5%, or greater, was cost-effective [27].

#### Diagnosis and management of LS

These studies investigated whether targeted genetic testing among CRC patients may be a cost-effective approach for the diagnosis and management of LS. Wang et al. [29] compared screening and no screening strategies for the diagnosis and management of LS, assessing that the screening approach (IHC followed by BRAF) was more effective than no screening but at higher cost [18–21, 25–29]. This study also confirmed the cost-effectiveness of universal screening of all patients with newly diagnosed CRC for LS [25, 27, 29]. Wang et al. evaluated cost-effectiveness of targeted genetic testing and surveillance programs in individuals at high-risk of Hereditary-nonpolyposis-CRC (HNPCC) vs unselective programs [30]. This analysis suggested that offering early targeted genetic testing and surveillance programs to young individuals at high-risk of HNPCC was a cost-effective strategy, if an improved compliance with recommended surveillance protocol was achieved in proven mutation carriers. Dihn et al. investigated whether primary screening by genetic testing for risk assessment in unaffected individuals was cost-effective as compared to practice based on clinical-risk criteria after malignancy was detected. Results showed how primary genetic screening for mutations in MMR genes in 25–35 years individuals with a risk-threshold of 5%, improved health outcomes among carriers and families, and was cost-effective relative to the common criterion of 47,438 €/QALY. From a cost-effective perspective, universal screening offered the greatest benefit in clinical outcomes at the least attractive cost-effectiveness ratios [27]. Ladabaum et al. estimated the cost-effectiveness of all strategies to identify and manage LS. Results showed that the systematic application of strategies to diagnose the LS among patients with newly diagnosed CRC could provide substantial clinical benefits at acceptable costs and that the cost-effectiveness of such testing depends on the participation rate among relatives at risk for the LS [28]. Specifically, among tumor-testing strategies, IHC followed by BRAF mutation testing was preferred, with an ICER of 34,345 €/LYG [21, 28, 29].

### KRAS

All studies included in this subgroup evaluated the cost-effectiveness of KRAS and BRAF mutation evaluation before therapy. In most of these studies, decision models were employed to assess the economic benefits associated to a predictive genetic testing for the subsequent treatment selection. Only one study compared different kind of KRAS mutation tests, with the assumption that the genetic testing effectiveness should depend also on the techniques accuracy. Inputs for the models were estimated using observations from randomized clinical trials (RCTs) and published literature. The majority of these evaluations explored the cost-effectiveness of several strategies, corresponding to different combination therapies with or without prior predictive testing. Blank et al. compared four strategies in order to assess the most cost-effective approach among them. Results showed that testing for BRAF and KRAS prior to Cetuximab was the most cost-effective approach, with an ICER of 62,653 €/QALY, despite high costs for predictive testing [31]. Vijayaraghavan et al. evaluated the cost-effectiveness of KRAS mutation testing throughout the comparison of 6 hypothetical therapy combinations for patients with mCRC in 2nd-line treatment, in Germany and United States [32]. The comparison showed how KRAS mutation testing is cost-saving at equivalent clinical outcomes. Behl et al. analyzed the cost-effectiveness of screening for KRAS and BRAF mutations combining four potential treatment combinations. This economic evaluation suggested that KRAS testing is cost-saving and the addition of BRAF testing may offer additional savings, with an ICER of 615,176 €/LYG [33]. Furthermore, Barone et al. showed that anticipating KRAS testing in patients with non-mCRC was economically sustainable. Indeed, ICER remains within the range of 6000–15,000 €/QALY, regardless of the level of risk of developing metastases [34]. To compare cost-effectiveness of different kind of KRAS mutation tests in patients with unresectable mCRC whose metastases are confined to the liver, Westwood et al. combined ten different methods for KRAS mutation testing [36]. The analysis showed that, under certain conditions, Therascreen KRAS RGQ PCR Kit was more cost-effective than pyrosequencing, even if there was a low accuracy among the evaluated strategies. The real cost-effectiveness of alternative combination therapies must be established comparing ICERs with the WTP-threshold of subjects to whom the model is addressed for. Three studies considered hypothetical WTP-thresholds based on estimation models [33, 35] or accepted by regulatory institutions [34], the latter, representing the recipients of the performed models.

### Prognosis

Alberts et al. compared cost-effectiveness of Oncotype DX vs standard care for CRC patients with stage II, T3, MMR-P (mismatch-repair-proficient). This analysis showed the cost-saving of the 12-gene assay on clinical aCT (adjuvant-chemotherapy) recommendations, with less toxicity risks and better results in terms of quality-adjusted survival [36].

### Summary of general characteristics of models and their adherence to CHEERS

Table 3 presents a summary of the methodological characteristics of the included studies. A wide range of simulation modelling approaches have been applied.

**Table 3:** Methodological characteristics of the included studies

Authors	Unit costs	Type of economic evaluation	Model	Model assumptions	Perspective	Time horizon	Discount rate (%)
Alberts et al. [36]	$, 2014	CUA	DAM, decision tree, Markov model	(a) In the absence of RCTs data, relative risk reduction was set according to the NCCN guidelines	Healthcare system perspective	Lifetime	3
Barone et al. [34]	€, 2012	CUA	DAM	(a) The level of risk of developing metastatic disease was set at the level of 1 patients out of 2, 1/3, 1/4, 1/5 and 1/10	Healthcare system perspective	NR	NR
Barzi et al. [17]	$, NR	CEA	Decision tree, Markov model	(a) The first-degree relatives of probands were considered as the healty individuals affected with LS who would be offered preventive measures	Societal perspective	Lifetime	NR
Behl et al. [33]	$, 2010	CEA	Markov model	(a) The difference in survivals among alternatives and the referent strategy (1) depends exclusively on a lack of response to (1)	Healthcare system perspective	10 years	3
Blank et al. [31]	€, 2010	CUA	Markov model	(a) A high number of assumptions related to the composition of the patient population	Healthcare system perspective	Lifetime	3
Dinh et al. [27]	$, 2009	CUA	Cohort simulation model	(a) Single-site testing only offered to FDRs of probands	Societal perspective	NR	3
Gallego et al. [18]	$, 2014	CUA	Decision tree	(a) Assumptions relying on the population involved in the model. Firstly, only FDRs have been considered, then universal screening has been evaluated	Healthcare system perspective	Lifetime	3
Gausachs et al. [19]	€, NR	CEA	Decision tree	(a) Assumptions related to the prevalence of germline mutation	Healthcare system perspective	NR	NR
Gould-Suarez et al. [20]	$, NR	CEA	Decision tree	Assumptions on the estimated model such as on the baseline prevalence of LS and other factors. All values were derived from published literature	Healthcare system perspective	NR	NR
Gudgeon et al. [21]	$, 2010	CEA	DAM	Assumptions on the estimated model such as on the baseline prevalence of LS and other factors. All values were derived from published literature	Healthcare system perspective	NR	NR
Ladabaum et al. [28]	$, 2010	CEA	Decision tree with Markov subtrees	Assumptions on the estimated model such as on the baseline prevalence of LS and other factors. All values were derived from published literature	Healthcare system perspective	Lifetime (or 100 years)	3
Leenen et al. [22]	€, 2013	CEA	DAM	(a) Assumptions made on uncertain parameters such as CRC risk for LS carriers, the method and risk reduction of LS surveillance and assumed adherence to LS surveillance programs	Healthcare system perspective	NR	3
Severin et al. [23]	€, 2012	CEA	DAM, decision tree, Markov model	Assumptions on the estimated model such as on the baseline prevalence of LS and other factors. All values were derived from published literature	Healthcare system perspective	Lifetime (or 120 years)	3
Sie et al. [24]	€, 2013	CEA	DAM, decision tree, Markov chain analysis	Assumptions on the estimated model such as on the baseline prevalence of LS and other factors. All values were derived from published literature	Healthcare system perspective	30 years	4
Snowsill et al. [26]	£, 2013/14	CUA	DAM, decision tree	(a) In the absence of RCTs data, estimates were sought from clinical experts	Healthcare system perspective	Lifetime (or 100 years)	3.5
Snowsill et al. [25]	£, 2013–14	CUA	Decision tree, individual patient simulation model	The rate of acceptance of a test was independent of any previous tests, and acceptance of one genetic test implied acceptance of all genetic testing	Healthcare system perspective	Lifetime	3.5
Vijayaraghavan et al. [32]	$ and €, 2009	CEA	Markov model	(a) Patients with KRAS mutant tumors received no benefit from EGFR inhibitors; (b) patients with KRAS mutant tumors received some benefit from combination therapy containing FOLFIRI or irinotecan; (c) KRAS mutation testing has a sentitivity of 95% and a specifity of 100%; (d) effectivess of cetuximab + FOLFIRI was equivalent to the effectiveness of cetuximab + Irinotecan	Healthcare system perspective	Lifetime	NR
Wang et al. [29]	$, 2010	CUA	DAM, Decision tree, Markov subtrees	Different clinical management programs and acceptance rates among probands and relatives until age 75 years based on their germline testing results and cancer risk were modeled	Healthcare system perspective	Lifetime	3
Wang et al. [30]	SGD, 2010	CEA	DAM, Decision tree, Markov subtrees	Related to compliance rates	Healthcare system perspective	Lifetime	3
Westwood et al. [35]	£, 2011	CUA	Decision tree, Markov model	(a) Assumption of equal prognostic value analysis for all tests for which information on technical performance was available from the online survey; (b) the differences between the outcomes of evaluated trials are exclusively caused by the different tests used; (c) test accuracy based on objective response can be compared with accuracy based on resection rates	Healthcare system perspective	Lifetime	3.5

*DAM* decision analytic model, *NR* not reported, *RCTs* randomized clinical trials, *NCCN* National Comprehensive Cancer Network, *LS* Lynch syndrome, *FDRs* first-degree-relatives, *CRC* colorectal cancer, *CEA* cost-effectiveness analysis, *CUA* cost-utility analysis

Many studies combined two or more modelling approaches [17, 23–26, 28–30, 35, 36], three studies used a Markov model [31–33], three a decision trees approach [18–20], three a decision analytic model [21, 22, 34] and one used a cohort simulation model [27]. Moreover, nine of the included studies were model-based CUAs, reporting QALY, the incremental cost per QALY [18, 25–27, 29, 31, 34, 35] and the cost-savings [36]. The other eight studies performed a CEAs reporting LYGs, the incremental costs per LYG [17, 22, 23, 27, 28, 30, 32, 33] and the incremental increase in no. of cases detected [19–21]. Most of the studies reviewed, performed economic evaluations from a healthcare system perspective [18–26, 28–36], while only two studies adopted a societal perspective [17, 27]. However, all of them included only direct medical costs. Furthermore, the majority of decision models have been performed adopting a lifetime horizon [17, 18, 23, 25, 26, 28–32, 35, 36]. One study used a 10-year time horizon [33] and another used a 30-year time horizon [24]. Contextually, most of the studies reported sensitivity analysis according to either probabilistic [18, 21, 23–26, 29, 31–33, 35, 36] and deterministic [17–19, 22, 25–27, 29–33, 36] methodology, in order to assess the robustness of model outcomes against parameter uncertainty and model assumptions. Scenario analyses were also performed, to analyze hypothetical future events by considering alternative potential outcomes [18, 23, 25, 26, 28–32, 34]. Across all included studies, the cost-effectiveness results were dependent on assumptions done regarding the sensitivity and specificity of tests. Table 4 presents the results of the assessment of adherence to the CHEERS guideline [12].

**Table 4:**
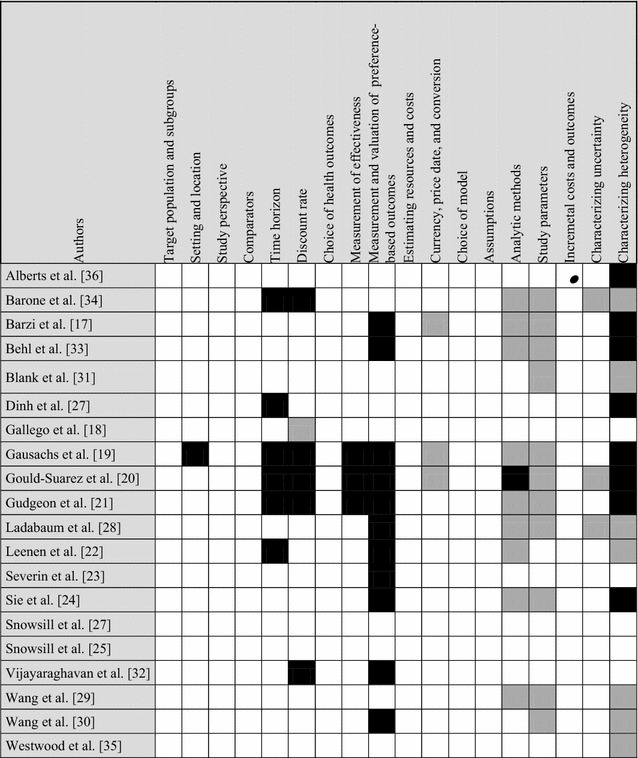
Evaluations of the simulation models on the basis of the CHEERS [12]

Color key: White: yes, black: no, grey: partially, ●: not applicable

*CHEERS* Consolidated Health Economic Evaluation Reporting Standards

Our analysis showed how studies generally did well in following the reporting guideline (even though the majority of articles did not explicitly state adherence to CHEERS or any other guideline). The main weaknesses of included studies relies on the reporting of the evaluation of heterogeneity through subgroup-specific results.

## Discussion

This work presents a systematic literature review of economic evaluations on genetic screening and targeted mutation detection tests used in CRC and published in the last 5 years. We included twenty studies divided into three main groups according to the issue addressed by each article: “diagnosis”, “treatment” and “prognosis” [17–36]. The main aim of these evaluations was to compare differences in costs related to health effects between alternative interventions in order to simplify and support the decision-making process. Several authors, through case and model-based studies, confirmed predictive genetic mutations’ clinical effectiveness, which generated an increase in their use in the clinical practice, with inevitable consequences in terms of costs [37–39]. Indeed, the latter caused concern about the reimbursement of these tailored-treatment as well as the need for adequate CEAs. In this scenario, CEAs of targeted therapies may represent a powerful tool to be used to evaluate the sustainability of the whole system. The present work contributes to the existing knowledge, by reviewing methods and approaches used in literature to evaluate the health economic impact of genetic testing. We observed an extensive use of scenario analysis to represent the multiple application of tests related to different patients’ group. It was also observed a wide use of sensitivity analyses in order to assess the robustness of the results regarding test acceptance and compliance. Our analysis also suggests how the accuracy of a diagnostic test, costs and assumptions done at the initial stage of the model estimation influence results of economic evaluation studies [37–39].

Regarding the evaluation of “diagnostic” approaches, all studies concluded that screening strategies for LS were cost-effective compared with no screening, with all of them finding at least one strategy that fell below a pre-specified threshold.

However, as previously discussed, given the different strategies and costs for each country, there was little consistency between results observed. In two studies, IHC with BRAF appeared to be the most cost-effective strategy [28, 29]. In further two studies, universal genetic testing was cost-effective compared to clinical risk criteria [27, 29]. Additionally, several authors agree that RBGs remain useful for screening CRC patients for LS, even if their limitation as a history-based tool and their relatively low sensitivity raise serious concerns about their effectiveness [17, 20, 23] (thus differing substantially from others [18, 19, 21, 22, 24–30]). Summarizing, all the studies suggested the cost-effectiveness of screening strategies in CRC patients, despite of age and in relatives in presence of LS.

Regarding “treatment”, the scenario appears little more complex and this clearly emerged from Behl et al. and Blank et al. studies [31, 33]. Indeed, both analyzed cost-effectiveness of KRAS and/or BRAF mutations’ screening test, obtaining considerably different results in terms of the amount of costs included in the model [31, 33].

Costs of base-case in the models performed by Blank et al. [31] were significant lower than costs calculated in the study of Behl et al. [33], probably due to the exclusion of resection costs, thus influencing models outputs (such as overall survival, QALY and ICERs). Both models showed that, for the lowest WTP-threshold, screening for KRAS and BRAF mutations is the most cost-effective approach among alternatives; KRAS-test represents the 2nd best choice and, finally, for the highest amount of WTP, administering anti-Epidermal Growth Factor Receptor (anti-EGFR) treatments to all patients might represent the best alternative in terms of clinical benefits. The latter hypothesis suggests that resources scarcity imposes the adoption of screening approaches in order to contain the high costs related therapies. Vijayaraghavan et al. and Barone et al., investigated cost-effectiveness of testing KRAS mutations before administering EGFR inhibitor [32, 34]. Despite they followed a different methodology approach, both studies showed cost-saving effects associated to the adoption of predictive testing to select patients for the following therapies. Of note, several recent RCTs underscored the importance of the evaluation of NRAS mutations (in addition to KRAS) before starting a treatment with anti-EGFR agents. Accordingly, only patients with both WT-KRAS and WT-NRAS (pan-WT-RAS) will benefit from the treatment and new CEAs evaluating the addition of this double test are therefore eagerly awaited. We further analysed economic evidence on targeted-mutation detection tests. Unfortunately, only one paper was found comparing different kind of KRAS mutation tests [35]. Results suggested that KRAS testing with Therascreen KRAS RGQ PCR kit (QIAGEN) was more costly and more effective than Pyrosequencing.

Our study has some limitations: first only English articles were included in our search. Furthermore, models heterogeneity as well as the strong dependence of economic evaluation on country-related settings, may affect the generalizability of our results. Lastly, a certain degree of subjectivity in our assessment should be taken into account, especially considering that is not feasible to summarize in details all elements reported in each article evaluated.

## Conclusion

Overall, economic evidence on genetic testing screening in CRC suggested that all the screening interventions evaluated in our systematic review are cost-saving for certain WTP-threshold. However, the poor evidence in this field, combined to the numbers of assumptions done to perform the models and to the lack of transparency and consistency in the methods used to derive costs, lead us a high level of uncertainty of the cost-effectiveness results provided in this study. For this reason, major research is required in order to assess the best combination among detection tests, type of genetic test screening and targeted-therapy. To overcome the major limitations found during this work, health providers, policy and decision makers should develop a common strategy on how models involving molecular testing should be structured and executed, in order to implement cross-comparable health economic evaluations and ensure an enlightened guidance in the development of standardized economic evaluations.

## Additional files

**Additional file 1: Table S1.** Search strategy.

**Additional file 2: Table S2.** Conversion of different currencies via PPPs (purchasing power parities) into Euro values.

**Additional file 3: Table S3.** Economic evaluation studies of interventions for diagnosis and treatment of colorectal cancer (2011–2016).

## Abbreviations

PM: personalized medicine
CRC: colorectal-cancer
WTP: willingness-to-pay
mCRC: metastatic CRC
USA: United States
IT: Italy
CH: Switzerland
ES: Spain
DE: Germany
UK: United Kingdom
Wal: Wales
SG: Singapore
MMR-P: mismatch-repair-proficient
LS: Lynch syndrome
FDRs: first-degree relatives
NR: not reported
NGS: next-generation-sequencing
RBG: Revised Bethesda Guidelines
DAM: decision analytic model
RCTs: randomized clinical trials
NCCN: National Comprehensive Cancer Network
CEA: cost-effectiveness analysis
CUA: cost-utility analysis
aCT: adjuvant chemotherapy
CE: cost-effectiveness
CRCP: colorectal cancer and polyposis
BSC: best supportive care
FH: family-history
QALY: quality-adjusted life year
ICER: incremental cost-effectiveness ratio
LYG: life years gained
MMR: mismatch-repair
ADRs: adverse drug reactions
NHSs: National Health Systems
CBA: cost–benefit-analysis
CHEERS: Consolidated Health Economic Evaluation Reporting Standards
IHC: immunohistochemistry
HNPCC: hereditary-nonpolyposis-CRC
anti-EGFR: anti-epidermal growth factor receptor
PPP: purchasing power parity
