# Supplementary material for: Personalized medicine in colorectal cancer diagnosis and treatment: a systematic review of health economic evaluations
Source: Cost Eff Resour Alloc. 2018 Jan 22;16:2. doi: 10.1186/s12962-018-0085-z (PMC5778687; doi:10.1186/s12962-018-0085-z)
Supplement: Supplementary file 1 — Additional file 1: Table S1. Search strategy. [file 12962_2018_85_MOESM1_ESM.docx]

| **Table S1. Search strategy** |  |
| --- | --- |
| **ELECTRONIC DATABASE** | **SEARCH STRATEGY** |
| Medline via Pubmed | **"Colorectal Neoplasms"[Mesh] AND ("Neoplasms"[Mesh] AND ("cost-benefit analysis"[MeSH Terms] OR ("cost-benefit"[All Fields] AND "analysis"[All Fields]) OR "cost-benefit analysis"[All Fields] OR ("economic"[All Fields] AND "evaluation"[All Fields]) OR "economic evaluation"[All Fields]) AND ("economics"[Subheading] OR "economics"[All Fields] OR "cost"[All Fields] OR "costs and cost analysis"[MeSH Terms] OR ("costs"[All Fields] AND "cost"[All Fields] AND "analysis"[All Fields]) OR "costs and cost analysis"[All Fields])) AND (("genomics"[MeSH Terms] OR "genomics"[All Fields] OR "genomic"[All Fields] OR "genome"[MeSH Terms] OR "genome"[All Fields]) OR ("genetic therapy"[MeSH Terms] OR ("genetic"[All Fields] AND "therapy"[All Fields]) OR "genetic therapy"[All Fields] OR "genetic"[All Fields])) AND ("0001/01/01"[PDAT] : "2016/08/31"[PDAT]) AND "humans"[MeSH Terms]**  **Other restrictions:**  **- Humans**  **- 2011-2016**  **- NOT: Addresses, Autobiography, Bibliography, Biography, Case reports, Comment, Dictionary, Directory, Duplicate Publication, Festschrift, Historical Article, Interactive Tutorial, Interview, Legal Cases, Legislation, News, Newspaper Article, Patient Education Handout, Periodical Index, Personal Narratives, Portraits, Video-Audio Media, Webcasts**  **- Search results: 119** |

­
