# Supplementary material for: Personalized medicine in colorectal cancer diagnosis and treatment: a systematic review of health economic evaluations
Source: Cost Eff Resour Alloc. 2018 Jan 22;16:2. doi: 10.1186/s12962-018-0085-z (PMC5778687; doi:10.1186/s12962-018-0085-z)
Supplement: Supplementary file 2 — Additional file 2: Table S2. Conversion of different currencies via PPPs (purchasing power parities) into Euro values. [file 12962_2018_85_MOESM2_ESM.docx]

| Table S2: Conversion of different currencies via PPPs (Purchasing Power Parities) into Euro values | | |
| --- | --- | --- |
| Country | year | PPP |
| Switzerland (Swiss Franc) | 2016 | 1,0728 Swiss Franc/€ |
| United Kingdom | 2016 | 0,8568 £/€ |
| USA | 2016 | 1,054 $/€ |

£ = Great Britain pounds, $ = US dollars, PPP = Purchasing Power Parities

Different currencies were converted to Euros using the following formula:

$$Amount € =\frac{Amount other currency}{PPP}$$
