# Supplementary material for: Personalized medicine in colorectal cancer diagnosis and treatment: a systematic review of health economic evaluations
Source: Cost Eff Resour Alloc. 2018 Jan 22;16:2. doi: 10.1186/s12962-018-0085-z (PMC5778687; doi:10.1186/s12962-018-0085-z)
Supplement: Supplementary file 3 — Additional file 3: Table S3. Economic evaluation studies of interventions for diagnosis and treatment of colorectal cancer (2011–2016). [file 12962_2018_85_MOESM3_ESM.docx]

| Table S3. Economic evaluation studies of interventions for diagnosis and treatment of colorectal cancer (2011-2016) | | | |
| --- | --- | --- | --- |
| Study | **Objective** | **Comparisons** | **Results** |
| Alberts et al. (2014) [36] | To evaluate aCTs recommendation changes with the 12-gene assay | (0) Oncotype DX+aCTs vs (1) aCTs recommendations | (0) was cost-effective vs (1) at least at the 95.5% |
| Barone et al. (2014) [34] | To evaluate CE of KRAS early testing in patients with CRC at risk of relapse and/or metastases | (1) Cetuximab+FOLFOX; (2) FOLFOX; (3) Bevacizumab+FOLFOX; (4) Cetuximab+FOLFIRI; (5) FOLFIRI | Anticipating KRAS-testing in patients with high-risk of relapse was cost-effective (ICERs of all comparisons remained within the WTP-threshold) |
| Barzi et al. (2015) [17] | To evaluate CE of screening testing to diagnose LS in the proband setting vs general population screening | 20 strategies compared with a referent strategy of no additional screening. (1) No screening; (2) Amsterdam+IHC+ germline; (3) Amsterdam+germline; (4) MMRpredict+IHC+germline; (5) MMRpredict+germline; (6) MMRpro+IHC+germline; (7) MMRpro+germline; (8) PREMM+IHC+germline; (9) PREMM+germline; (10) RBG+IHC+germline; (11) RBG+germline; (12) IHC+germline; (13)IHC+BRAF+germline; (14) MSI+germline; (15) IHC+BRAF+germline; (16) MSI+germline; (17) MSI+IHC+germline; (18) MSI+IHC+BRAF+germline; (19) Up-front germline; (20) PREMM-GP screening | The most cost-effective strategy was (6), meeting also the WTP-threshold |
| Behl et al. (2012) [33] | To assess the CE of screening for KRAS and BRAF mutations before EGFR inhibit treatment | (1) No anti-EGFR therapy no screening; (2) anti-EGFR therapy no screening; (3) KRAS mutation screening; (4) KRAS and BRAF mutation screening; | (4) is preferable for WTP-threshold<1 million $/QALY; with an increasing in WTP (3) and (4) are preferred. (2) is the most preferred for WTP>3million $/QALY. None of the comparisons met the set WTP-threshold |
| Blank et al. (2011) [31] | To assess the CE of testing for KRAS and BRAF mutations before EGFR inhibit treatment | (0) No cetuximab (BSC) (reference strategy); (1) KRAS mutation testing and then cetuximab + BSC; (2) KRAS mutation testing with subsequent BRAF testing of KRAS wild-type patients and then cetuximab + BSC; (3) cetuximab without testing + BSC. | (1) is preferable for 10,000<WTP< 40,000 €/QALY; (2) is preferable for 40,000< WTP<100,000 €/QALY |
| Dinh et al. (2011) [27] | i) To assess CE of primary screening and managing for LS in unaffected persons; ii) To compare different algorithms in selecting high-risk CRC patients eligible for genetic testing | (a) Screening strategies compared with current practice; (b) Comparisons among screening strategies; (c) Comparisons among screening strategies under assumptions. (1) Strategy 1: Universal primary genetic screening starting at age 20 vs current practice; (...); (20) Strategy 20: Universal primary genetic screening starting at age 40 vs current practice | Primary genetic screening for MMR mutations was the most cost-effective strategy, meeting also the WTP-threshold |
| Gallego et al. (2015) [18] | To evaluate CE of NGS panel testing in the diagnosis of LS | NGS vs standard care. (0) IHC+BRAF; (1): only LS genes; (2): (1)+genes associated with autosomal dominant CRCP syndromes with high penetrance of colorectal cancer; (3): (2)+genes associated with autosomal recessive CRCP syndromes with high penetrance of colorectal cancer; (4): (3)+genes associated with autosomal dominant CRCP syndromes with low penetrance of colorectal cancer | There is a 99% probability that NGS was cost-effective compared to standard care |
| Gausachs et al. (2012) [19] | To evaluate CE of using Methylation in the diagnosis of LS | (1) BRAF vs MLH1 mutation analysis: BRAF V600E testing for all individuals, plus eventually MLH1 mutation testing; (2) Hypermethylation vs MLH1 mutation analysis: MHL1 Hypermethylation testing for all individuals, plus eventually MLH1 mutation testing; (3) MLH1 mutation analysis: MLH1 mutation testing for all individuals | (2) was the most cost-effective strategy |
| Gould-Suarez et al. (2014) [20] | To evaluate the CE of 10 strategies for the diagnosis LS | (1) RGB+MMR4; (2) RGB+IHC4; (3) RGB+MSI+IHC4; (4) RGB+MSI+MMR4; (5) MMR4; (6) MSI+IHC4; (7) MSI+MMR; (8) IHC4+MSI+MMR; (9) IHC4/MSI; (10) IHC/MSI | (10) was cost-effective vs (6) and (7) |
| Gudgeon et al. (2011) [21] | To assess the CE of strategies to identify LS, focusing only on tumor-based protocols that begin with IHC testing | IHC-first screening approach of all CRC cases followed by different subsequent screening tests (BRAF, Seq-Rearr, methyl test). (1) IHC direct to sequencing; (2) IHC with BRAF; (3) IHC with methyl; (4) IHC with BRAF then methyl; (5) IHC with methyl then BRAF | (2) was the most cost-effective option |
| Ladabaum et al. (2011) [28] | To investigate CE of strategies to identify and manage LS | (1) Referent strategy of no test; (2) Several clinical criteria strategies: (2a) Amsterdam/IHC; (2b) Amsterdam/germline; (2c) MMRpredict/IHC; (2d) MMRpredict/germline; (2e)MMRpro/IHC; (2f) PREMM/IHC; (2g) RBG/IHC; (2h) MMRpro/germline; (2i) PREMM/germline; (2l) RBG/germline; (3) Several tumor-testing strategies: (3a) IHC; (3b) IHC+BRAF; (3c) MSI; (3d) MSI+IHC; (3e) MSI+IHC+BRAF; (4) Up-front germline testing | Among tumor-testing strategies, (3b) was the preferred, meeting also the WTP-threshold |
| Leenen et al. (2016) [22] | To assess the CE of routine LS screening | (0) LS screening for patients ≤50 years; (1) LS screening for patients ≤60 years; (2) LS screening strategy for patients ≤70 years; (3) RBG | (2) was the most cost-effective strategy, meeting also the WTP-threshold |
| Severin et al. (2015) [23] | To analyze the CE of different LS screening | 22 testing strategies considering family-history assessment, analysis of tumor samples (IHC, MSI, BRAF mutation testing) and genetic sequencing were analyzed. (0) No screening, (1) Counseling including RBG+IHC+BRAF+sequencing, (2) Counseling+IHC+BRAF+sequencing, (3) Counseling+direct sequencing (...) | (0) has a probability of 87% of being considered cost-effective |
| Sie et al. (2014) [24] | To evaluate CE of raising the age limit for CRC tumor genetic testing from 50 to 70 years | 1. CRC ≤70 vs (2) CRC ≤50; | (1) was the most cost-effective option, meeting also the WTP-threshold |
| Snowsill et al. (2015) [26] | To estimate the cost-utility of strategies to identify LS | (1a) No testing; (1b) Amsterdam II criteria for diagnosis (FH); (2) IHC4+mutation testing; (3) IHC4+BRAF+mutation testing; (4) MSI+mutation testing; (5) MSI+BRAF+mutation testing; (6) MSI+BRAF+mutation testing and IHC; (7) IHC4+mutation testing+MSI+BRAF; (8) Direct mutation testing | At the set WTP-threshold, (5) was the most cost-effective strategy |
| Snowsill et al. (2014) [25] | To assess CE of routine LS screening strategies | (1) No testing; (2) IHC followed by genetic testing; (3) IHC followed by BRAF and genetic testing; (4) MSI test followed by genetic testing; (5) MSI test followed by BRAF testing and genetic testing; (6) MSI test followed by BRAF, IHC and genetic testing; (7) IHC test followed by MSI, BRAF and/or genetic testing; (8) Universal genetic testing | (5) was the most cost-effective option |
| Vijayaraghavan et al. (2012) [32] | To assess the CE of testing KRAS mutations before administering EGFR inhibitors | (1) Combination therapy (cetuximab+irinotecan/FOLFIRI) with KRAS mutation testing; (2) combination therapy (cetuximab+irinotecan/FOLFIRI); (3) cetuximab with KRAS mutation testing; (4) cetuximab; (5) panitumumab with KRAS mutation testing; (6) panitumumab | Using KRAS testing to restrict use of anti-EGFR to patients with KRAS wild-type tumors was cost-saving in both USA and Germany |
| Wang G. et al. (2012) [29] | To assess CE of strategies to identify and manage LS | (1) No testing; (2) several clinical criteria strategies; (3) Several tumor-testing strategies; (4) Up-front germline strategies; (5) Up-front germline testing. | The study showed the cost-effectiveness of universal screening of all patients with newly diagnosed CRC for LS |
| Wang V. et al. (2012) [30] | To evaluate, with consideration of varying compliance rates, the long-term CE of targeted genetic testing and surveillance programs in identifying and managing LS | (a) Genetic testing+targeted surveillance programs; (b) Unselective intensive surveillance programs with no genetic testing | 1. is genetic dominant vs (b) |
| Westwood et al. (2014) [35] | To compare CE of different kind of KRAS mutation tests | (1) Cobas KRAS Mutation Test Kit (Roche Molecular Systems); (2) Therascreen KRAS RGQ PCR Kit (QIAGEN); (3) Therascreen KRAS Pyro Kit (QIAGEN); (4) KRAS LightMix Kit (TIB MOLBIOL); (5) KRAS StripAssay (ViennaLab); (6) HRM analysis; (7) Pyrosequencing; (8) MALDI-TOF mass spectometry; (9) NGS; (10) Sanger sequencing | For the set WTP-threshold, (7) is the preferred test |
| aCT: adjuvant chemotherapy, CE: cost-effectiveness, CRC: Colorectal cancer, CRCP: colorectal cancer and polyposis, LS: Lynch Syndrome, NGS: Next-Generation-Sequencing, BSC: Best Supportive Care, FH: Family-history, RBG: Revised Bethesda Guidelines, QALY: Quality-adjusted life year, WTP: Willingness-to-pay, ICER: Incremental cost-effectiveness ratio, USA: United States, MMR: mismatch-repair. | | | |
